# Visual gaze bias motion detection by split eyes in miniature whiteflies

**DOI:** 10.1016/j.isci.2025.112730

**Published:** 2025-05-22

**Authors:** Tomer Urca, Fritz-Olaf Lehmann

**Affiliations:** 1 Department of Animal Physiology, Institute of Biosciences, University of Rostock, 18059 Rostock, Germany

**Keywords:** Sensory neuroscience, Cognitive neuroscience

## Abstract

Vision in miniature insects is constrained by an extremely small number of ommatidia and brain cells available for image processing. Here, we explore how one millimeter whiteflies cope with these limits during vision-mediated locomotion by linking micro-tomographical reconstructions of the eye to changes in visual gaze in maneuvering flight. The split eye design accommodates two flat arrays with 31 and 42 ommatidia at 53° mean angular spacing, which limits panoramic view. Low optical resolution with 14.4° interommatidial angle hampers object recognition and visual motion detection needed for body posture stability reflexes. During maneuvering, mean gaze direction of both eye sections differs in elevation and azimuth depending on yaw, pitch, and roll angles. Dorsal and ventral eye sections thus receive visual information from specific areas in the visual environment. Collectively, splitting the eye into separate ommatidia arrays potentially allows whiteflies to maintain elaborate vision-controlled flight behaviors even at reduced visual capacity.

## Highlights

• Split compound eyes suggest optical limits of vision in miniature whiteflies
• Interommatidial angle of 14.4° hampers object recognition and visual motion detection
• Changes in body posture produce different optic flow patterns on each eye section
• Split eyes might improve vision-controlled flight at reduced visual capacity

## Graphical abstract

**Figure:**
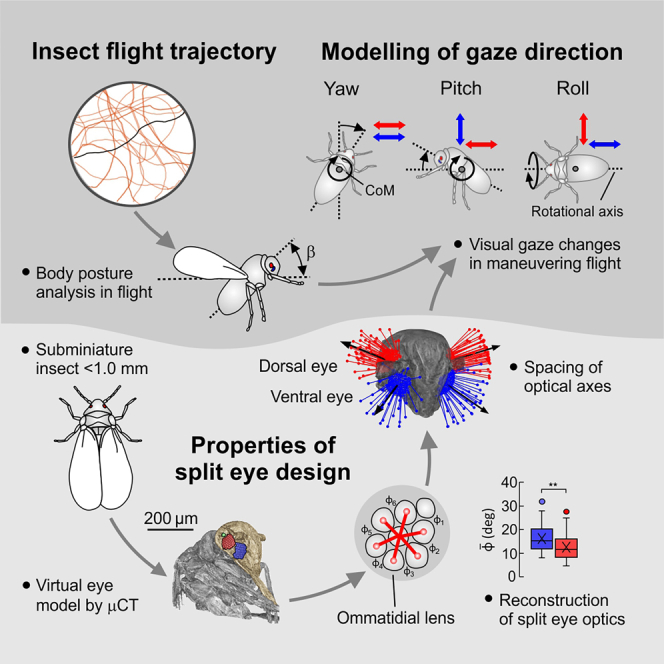

## Introduction

The visual system of flying insects serves several functions, providing essential information for orientation and navigation in complex three-dimensional environments, compensatory flight course control, and posture-stability reflexes.^1^^,^^2^^,^^3^^,^^4^^,^^5^^,^^6^^,^^7^^,^^8^^,^^9^^,^^10^ Numerous electrophysiological and behavioral studies have demonstrated the important role of visual object recognition and visual motion detection for the aforementioned behaviors.^1^^,^^11^^,^^12^^,^^13^^,^^14^ The visual capacity of the insect compound eye depends on several components, in particular on its optical properties such as the light opening angle of the ommatidia and angular spacing of their visual axes,^15^^,^^16^^,^^17^ and the neural power to process visual information by the brain. To minimize angular spacing, for example, predatory insects like robber flies and dragonflies exhibit optical “acute zones” with ommatidia orientated in the forward direction that are similar to the vertebrate fovea.^15^ The reduced eye curvature and large lens diameters in flattened acute zones lead too small separation angles with elevated optical resolution and signal-to-noise ratios, respectively, but at the cost of a detailed panorama view.^18^^,^^19^^,^^20^^,^^21^^,^^22^^,^^23^ Thus, acute zones prioritize sections of the visual environment around the animal and their occurrence is a common phenomenon in compound eyes.^24^

A decrease in body size leads to increasing constraints on the optical apparatus that requires a simplification of the insect eye.^25^^,^^26^^,^^27^^,^^28^ This includes a decrease in the number of ommatidia, smaller facet diameters, and reduced ommatidium lengths. A reduced number of ommatidia does not necessarily limit the insect’s field of view, as large interommatidial angles only reduce optical resolution. Dismounting the number of ommatidia is thus common in small insects leading to reduction factors of up to ∼60-fold in Chalcids.^27^ In the smallest known insect species, *Megaphragma mymaripenne*, the eye contains only 30 ommatidia on each body side.^27^ Size reduction of each ommatidium further attenuates the insect’s visual capacity. Small corneal lenses and apertures cause elevated light diffraction and limit the number of photons available to the rhabdomers.^29^ As a consequence, small eyes have lower signal-to-noise ratios at the level of the photoreceptors including their downstream neurons.^30^ Both factors, light diffraction and low signal-to-noise ratio, attenuate the eye’s resolving power and thus the detectability of small visual objects at low light conditions. In addition to optical constraints, in small insects below ∼300 μm body length,^31^ the brain may contain less than ∼1,000 cells compared to larger insects such as, for example, the fruit fly with ∼135,000 cells and honeybees with ∼1,000,000 cells.^32^^,^^33^ As visual motion detection requires a complex neural network (elementary motion detector) residing in the visual ganglia,^11^^,^^14^^,^^34^^,^^35^^,^^36^ a substantial reduction in brain size is thought to be correlated with a reduced capacity to process sensory information and initiate behavioral programs.^27^ It is widely unknown how miniaturized insects cope with such constraints, while keeping their motor programs for visually mediated flight behaviors. In particular, a reduction in visually capacity is thought to hamper the detection of self-induced optic flow during body translation and rotation and thus the information needed for body posture stability reflexes.^37^^,^^38^ This holds, in particular, for flying insects without halteres.^39^^,^^40^ Besides few examples, our knowledge on vision in insects with bodies in the sub-millimeter range is poor and the question of how moving miniature insects circumvent adverse effects of miniaturization widely unexplored.

The one millimeter silverleaf whitefly *Bemisia tabaci* (Hemiptera: Aleyrodidae) is one of the few examples in which flight behavior^41^^,^^42^^,^^43^ and the spectral sensitivity of the visual system is studied in greater detail.^44^^,^^45^^,^^46^ Unlike in larger insects, the compound eye of the whitefly is divided into a dorsal and ventral substructure.^47^ Although a previously published study reported subtle differences in spectral sensitivity between both substructures,^48^ the exact purpose of the division is still unclear. In general, few explanations exist on the function of split eyes such as on the over and under water eyes of whirligig beetles^49^ and, most prominently, the turbinate eye of male mayflies.^50^ It has been suggested that their functional separation is akin to acute zones allowing the insect to simultaneously direct its visual gaze on two areas of interest, but no detailed data exist that prove this idea.^50^ The latter is also due to the lack of knowledge on the optical properties of miniaturized facets and neural network processing visual information. We hypothesize that the split eye of whiteflies is an adaptation that mitigates the adverse effects of miniaturization by fulfilling a dual purpose, i.e., creating ommatidia arrays similar to acute zones found in larger insects and, second, simplifying visual motion detection by separating the eye into horizontal and vertical sensitive zones for optic flows.

Here, we tested the above hypothesis combining two approaches: by an estimation of the eye’s optical properties from micro-computed tomography measurements and behavioral experiments on freely and tethered flying whiteflies. Tomography shows that dorsal and ventral eyes have different number of facets, facet diameter, lens curvature, and focal length. The two ocelli of the animal are not investigated. The flight data revealed unexpected changes in visual gaze with changing body pitch angle and thus during flight speed control. While the changes in gaze of the ventral eye mainly occurred in the vertical direction (elevation), gaze of the dorsal eye was opposite and mainly changed in the horizontal direction (azimuth). Gaze estimations at changing body roll yielded a reverse effect with gaze changes of the ventral eye changing largely in the horizontal and that of the dorsal eye in the vertical direction.

## Results and discussion

### Eye structure

**Figure 1:**
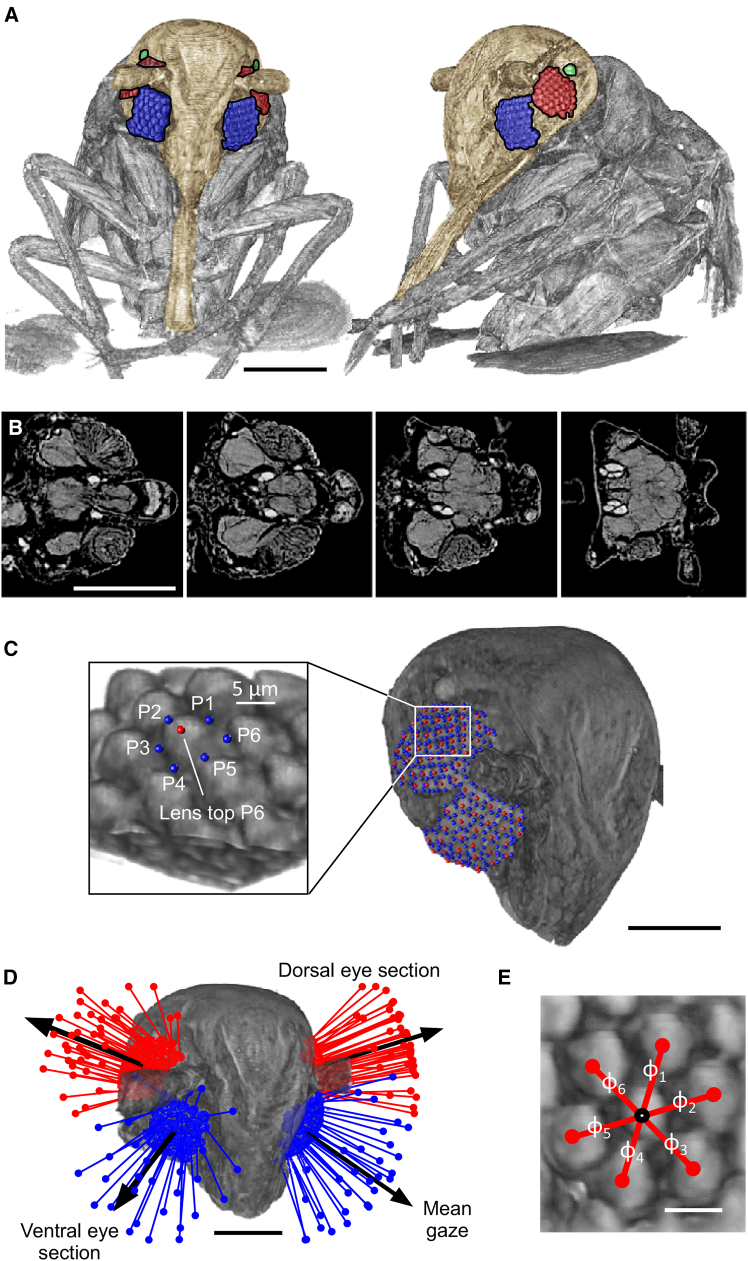
Micro-tomographic reconstruction, main visual axes, and ommatidial spacing in whiteflies (A) Lateral (left) and frontal (right) view of a female *Bemisia tabaci*. The compound eye is subdivided into a dorsal (red) and a ventral (blue) section of the head capsule (brown). There is one large ocellus (green) on each body side. (B) Cross-sections (top view) through the head capsule at 95 μm, 80 μm, 70 μm, and 50 μm depth from the dorsal outer cuticle (left-to-right). (C) Opening area of each corneal lens is estimated from 6 points (P1-P6, blue) and main optical axis from the line connecting the tip of the lens (red, P6) and the midpoint of the circular lens basis. (D) Three-dimensional orientation of visual axes of all ommatidia on the ventral (blue) and dorsal (red) eye sections. Mean gaze of dorsal and ventral eye sections are shown as black arrows. (E) Mean interommatidial angle of a single ommatidium is derived as mean angular difference between its visual axis (center point) and the visual axes of the six surrounding facets (ϕ_1_ - ϕ_6_). Scale bars are 100 μm in (*A* and *B*), 50 μm in (*C* and *D*), and 5 μm in (*E*).

**Figure 2:**
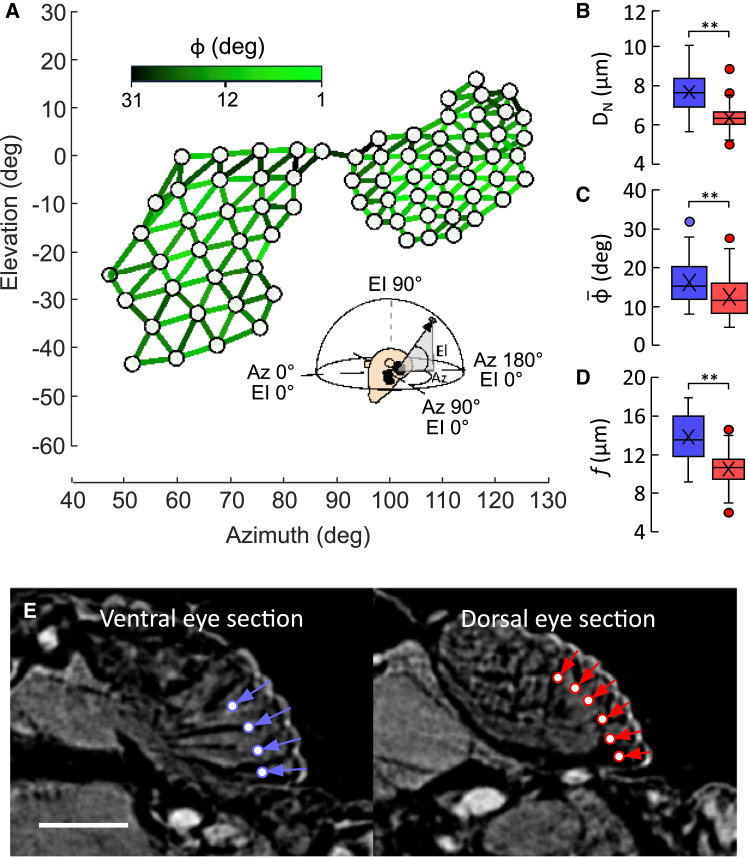
Optical properties of corneal lenses and visual gaze (A) Mean orientation of visual axes of all ommatidia of the right eye of 3 female and 3 male whiteflies (white dots). Orientation is plotted in spherical coordinates as vertical elevation (EL) and horizontal azimuth (Az) of a virtual sphere surrounding the head capsule (inset). Angles are shown for an animal at zero body pitch angle (longitudinal body axis in the horizontal). Interommatidial angle, ϕ, is plotted as a connecting line in pseudo-color. (B and C) (B) Mean cornea diameter of both eye sections and (C) mean absolute interommatidial angle (*n* = 250 ommatidia, dorsal eye section, red; *n* = 189 ommatidia, ventral eye section, blue). (D) Mean optical focal length (*n* = 86 ommatidia, dorsal eye section, red; *n* = 64 ommatidia, ventral eye section, blue). (E) Micro-CT images of longitudinal sections of ommatidia and estimated focal point of the cornea lenses (white points) inside the rhabdomere of dorsal (red) and ventral eye (blue) sections. Scale bar is 30 μm. Data in (B*–*D) are shown as boxplots with means (cross), medians (horizontal line), quartiles (hinges), and whiskers extending to the most extreme data point no further than 1.5 times the interquartile range away from the box. Outliers are plotted as circles. ∗∗, statistical significance at *p* < 0.01.

From micro-computed tomography (micro-CT)^16^ we derived the three-dimensional head ultrastructure of male and female miniature whiteflies *Bemisia tabaci* including dorsal and ventral sections of the split compound eye (Figures 1A–1C). As specimens were subjected to a preparation process prior to scanning, we cannot rule out certain effect of tissue deformation on the measured optics (cf. Methods section). Head width of males is 116 ± 8.81 μm and significantly smaller than the 126 ± 3.45 μm of females (t test, *p* < 0.001). We find that each eye consists of 73 ± 2 ommatidia (*N* = 6 animals), divided into two arrays of 42 ± 2 and 31 ± 1 ommatidia on the dorsal and ventral side, respectively (t test, *p* < 0.001). Ommatidia number is not correlated with head width or sex (t test, *p* > 0.91 for both). For comparison, the ∼3 mm fruitfly *Drosophila melanogaster*^51^ has 782 ± 17 and the ∼1.5 mm fungus gnat *Neoplatyura modesta* 303 ommatidia on each body side.^16^ To determine the geometric properties of the outer optical structures, we estimated diameter and radius of curvature of the corneal lenses of each ommatidium, and its orientation of the mean visual axis (gaze vector, Figures 1D and 2A). Mean lens diameter of the dorsal eye section is ∼6.35 ± 0.51 μm (*n* = 250 ommatidia) i.e., ∼17% smaller than the lenses of the ventral section (∼7.67 ± 0.89 μm, *n* = 189; generalized linear model, *p* < 0.001; Figure 2B). Lens diameter among both sexes slightly increases with increasing head width (generalized linear model, *p* < 0.001), thus females have larger lenses, but is not correlated with sex when normalized to head width (generalized linear model, *p* = 0.65). Mean lens base area (optical aperture) of the two sections amounts to ∼31.8 ± 5.19 μm^2^ and ∼46.8 ± 10.76 μm^2^, respectively. The latter data mean that the ventral eye lenses collect ∼36% more light than those of the dorsal eye. The size of the whitefly’s lens facet is almost identical to the 8.1 ± 0.3 μm diameter lens reported for the smallest hymenopteran species *Megaphragma mymaripenne* (body length, 0.2 mm).^52^ However, whiteflies are ∼5 times larger (body length, 0.90 ± 0.18 mm, *N* = 16 animals) than *M. mymaripenne* suggesting a lower size limit for a single ommatidium in insects. Although the small aperture reduces photon collection, this effect is mitigated by a decrease in focal length of the corneal lens (see paragraph below, Figure 2D). Strongly curved sphere-like lenses are typical for many miniature insects^25^^,^^52^^,^^53^ leading to round rather than the classic hexagonal-shaped flat lenses seen in larger insect.^54^^,^^55^^,^^56^ Flattening of substructures in the eyes of whiteflies improves the optical resolution within the field of view. In this regard, the dorsal eye section yields ∼23% higher optical resolution than the ventral eye section as mean interommatidial angle between neighboring ommatidia is ∼12.5 ± 4.43° (*n* = 250 ommatidia) and ∼16.2 ± 4.64° (*n* = 189 ommatidia; generalized linear model, *p* < 0.001), respectively (Figures 1D, 1E, 2A, and 2C). Within each eye section, the interommatidial angle was smallest at the center amounting to ∼1° and increased toward the edges of up to ∼30°. In *Drosophila*, the angle varies only little and amounts to ∼4° in all directions.^57^

Mean outer curvature radius of the facet lenses differs by ∼31% between both eye sections, i.e., 3.98 ± 0.72 μm (*n* = 86 ommatidia) for the dorsal and 5.81 ± 0.92 μm (*n* = 64 ommatidia; generalized linear model, *p* < 0.001) for the ventral eye section. The radius increases with increasing head width (generalized linear model, *p* = 0.01) but is independent of sex when normalized to size (generalized linear model, *p* = 0.15). As our micro-CT images did not allow us to clearly estimate the inner lens radius, we only considered light refraction at the transition from air to the corneal side of the lens with a lens refraction index of 1.4.^25^ The aforementioned lens radii translate to a focal length of ∼10.5 ± 1.76 μm (*n* = 86) and ∼13.8 ± 2.33 μm (*n* = 64 ommatidia), respectively (Figure 2D). The ∼24% difference in focal length suggest different depths of field and magnifications in both eyes sections. Figure 2E shows that the depth of the focal point within the rhabdomere is ∼31% and ∼40% mean ommatidia length (∼34 μm), respectively. Similar values are reported for the much bigger housefly *Musca domestica* with a focal point at ∼57% ommatidia length.^58^^,^^59^

### Flight speed, body pitching, and head movements

**Figure 3:**
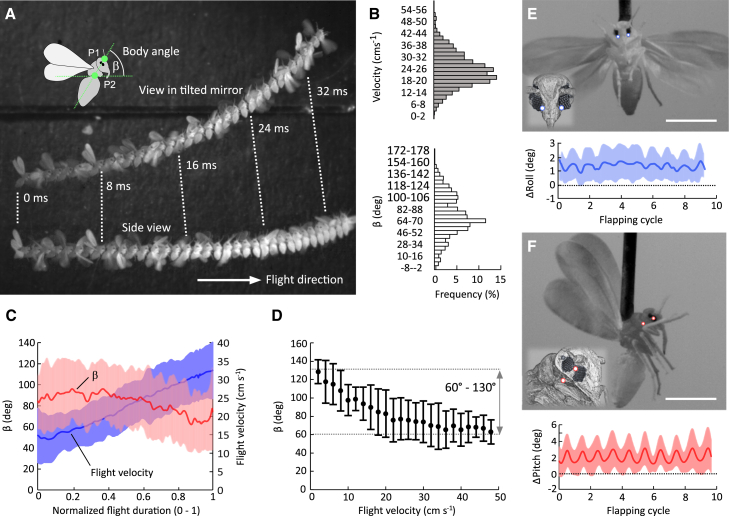
Body and head orientation during free and tethered flight (A) Flight maneuver of a freely flying whitefly. Flight direction is from left to right. The image is generated by superimposing video frames sampled every 1.0 ms. The lower trace shows the animal from side while the upper trace is the view by a 65° tilted mirror. Body pitch angle with respect to the horizontal was estimated from the line connecting head tip and the transition between abdomen and thorax (green, inset). (B) Frequency of forward flight velocities (upper) and body pitch angle β (lower histogram). (C) Mean temporal change in forward velocity (line, red) and body pitch angle (line, blue) with colored areas denoting standard deviations. (D) Body pitch angle plotted as a function of binned forward flight velocity (bin width 2.0 cms^−1^; means ± standard deviation). Flight sequences stem from *N* = 21 animals in (*C* and *D*). (E and F) (E) Changes (Δ) in mean head roll (frontal view) and (F) head pitch angle (side view) with respect to the fixed body and estimated from lines connecting two markers (white points) of the tethered flying animal. Colored area shows standard deviation of the mean (*N* = 12 animals). Scale bars are 500 μm.

To determine the changes in orientation of the visual axes of each facet in a natural behavioral context, we measured body posture orientation during free flight from the longitudinal body axis with respect to the horizontal. The animals voluntarily took off from a small post inside a flight chamber (Figure 3A). To study the effect of body pitching on flight speed control, we analyzed flight bouts in which the animals flew straight without turning. Data show that flight duration varies between ∼22 ms and ∼69 ms, at ∼37 ± 16 ms (*N* = 21 flight sequences) mean flight time in each bout. Typical horizontal forward speed is ∼23.8 ± 8.6 cm s^−1^ but covers flight speeds from hovering to ∼53 cm s^−1^ maximum speed (Figure 3B). The latter value converts into ∼576 body lengths s^−1^. For comparison, high-performance predatory flies *Holcocephala fusca* and *Coenosia attenuata* fly at an average speed of 120 and 175 body lengths s^−1^ when chasing artificial targets^60^ and *Drosophila* flies at ∼400 body lengths at 1.2 ms^−1^ maximum flight speed.^61^ Figures 3B–3D shows that body pitching angle in whiteflies decreases with increasing forward speed, suggesting that the inclination of total flight force vector redirects aerodynamic forces for thrust control. Once reaching stable forward flight following take-off, the animals achieved a flight speed of ∼27.5 ± 11.1 cm s^−1^ at a body pitch angle of ∼48.1° ± 16.1°. While flying in the attempt to land, whiteflies systematically reduce flight speed down to ∼17.8 ± 6.8 cm s^−1^ at a body pitch angle of ∼113° ± 26.7° (Figures 3C and 3D).

To consider the contribution of head motion to visual gaze, we scored head motion in tethered flying whiteflies by scoring the relative orientation of two morphological body markers (Figures 3E and 3F). We found only subtle changes in head roll and pitch angles of typically not more than ∼1.0° peak-to-peak amplitude. Changes in pitch are synchronized with the flapping cycle and likely result from the jitter of the thoracic marker due to thorax deformation during wing motion. Mean angular changes during flight are ∼0.95 ± 0.72° (*N* = 12) for head rolling and 1.88 ± 1.64° (*N* = 12 animals) for head pitching. Altogether, the data suggest no capacity of whiteflies to move their heads independently of the thorax and thus visual gaze solely depends on body posture.

### Direction of visual gaze

**Figure 4:**
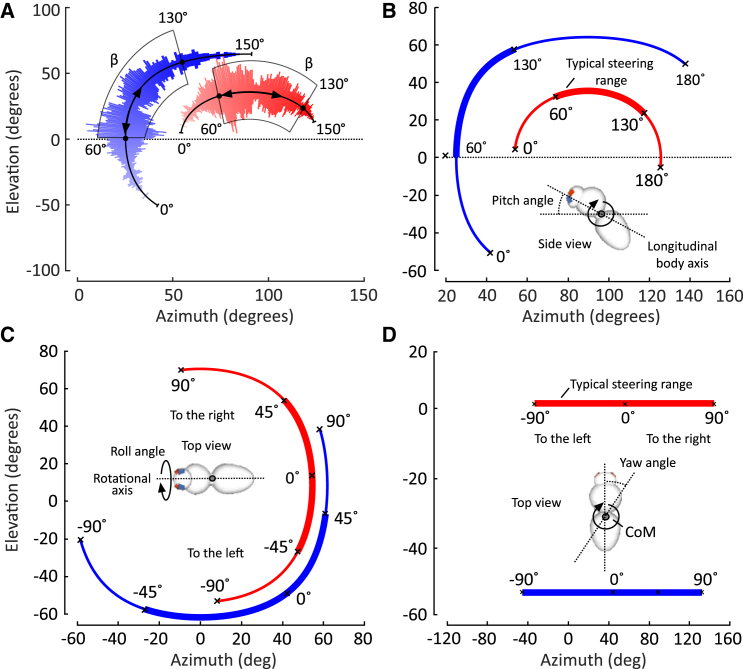
Gaze changes with changing body angles (A) Mean gaze of freely flying whiteflies of dorsal (red) and ventral (blue) eye sections is plotted in spherical coordinates for changes in body pitch angle β and ignoring body translation. Pitch angle is plotted as pseudo color running from 0° (pale) to 150° (strong). Frequency of β (cf. data in Figure 3B) is shown as bar length and maximum bar length corresponds to 480 data points in a 1° bin of β. Highlighted β between 60° and 130° contain 75% of all measured pitch angles. (B–D) Mean gaze of the dorsal and ventral eye sections for typical ranges of pitch, roll and yaw angles during maneuvering flight (excluding body translation). Insets show the axes of rotation about the animal’s center of mass (CoM).

To estimate visual gaze of both eye sections, we extended the optical axis of the facets and estimated azimuth and elevation angles from the point of passage of the axes on a virtual sphere surrounding the head. The sphere had a 10 mm radius, thus azimuth and elevation are broadly independent from the exact point of rotation inside the animal during body pitching. The coordinate origin of the sphere is between the left and right converging point of all visual axes on each body side. We found that mean visual gaze (cf. Methods section) differs by ∼53° between dorsal and ventral eye sections (Figure 1D) and changes with changing body pitch angle between 0° and 150° during flight (Figure 4A). Surprisingly, we also found that mean visual gaze of both eye sections behaves differently in response to changes in body pitch angles during flight speed control. Within the typical range of body pitch angles (60°–130°, Figures 4A and 4B), gaze of the ventral eye section strongly varies between ∼0° and ∼60° in elevation but little in azimuth. Conversely, mean visual gaze of the dorsal eye section barely varies in elevation while azimuth strongly increases from ∼75° to ∼117° with increasing body pitch. We also numerically estimated mean gaze for changing body roll and yaw within typical ranges of angles reported for small freely flying insects (∼±90° for yaw,^61^ ±30° – ±50° for roll^62^). The data suggest that body rolling yields an opposite effect to pitching with elevated/less changes in azimuth/elevation in the ventral eye, and less/elevated changes in the dorsal eye section (Figure 4C). As expected, changes in body yaw produce only changes in retinal optic flow in the horizontal and no optic flow in the vertical direction (Figure 4D). The distinct patterns of angular sensitivity suggest that both eye sections respond quite differently in flight during both self- and externally induced rotations about the three body axes. Thus, the different activation patterns of the retina by optic flow might help the insect to distinguish between yaw, pitch, and roll rotations. This could potentially be achieved by comparing the strength of visual stimulation between both eye sections, which in turn may lead to enhanced posture and flight course stability in the miniature insect.

### Concluding remarks

Miniaturization of an animal leads to considerable challenges, hampering almost all organs and structures as outlined previously.^27^^,^^28^^,^^31^^,^^63^ The smallest insect species, the ∼139 μm wingless egg parasitoid wasp *Dicopomorpha echmepterygis*, is one of the smallest metazoans in nature with a size similar of an ameba.^27^^,^^63^ Compared to a single cell, however, flying insects have wings, a muscle system and brain cells suited for navigating, orientating, and stabilizing their body during flight. Understanding how nature simplifies and reduces structures that are necessary for these behaviors is central to our understanding of how miniaturization works during evolution. Our data on whiteflies reveal the changes made to the visual system to overcome the limits of miniaturization, i.e., split eyes to create flat ommatidia arrays akin to an acute region for better visual resolution, reduction in number of ommatidia and the increase of corneal lens opening angles for higher rates in photon flux. The split eye structure may link the reduced visual capacity with the expected low power of neural processing, representing a potential solution to how tiny insects might maintain some of their sensorimotor capacity needed for flight. This includes various behaviors, such as object fixation behavior and optomotor responses. However, we cannot rule out that the split into two ommatidia arrays with different optics may be related to other anatomical constraints or to segregation of visual roles of different environmental light conditions in different viewing angles. Besides visual transmission and neural processing by small brains, miniature insects are also model systems for aerodynamics and energetics of wing flapping at elevated air viscosity and thus represent a group of animals suitable for integrative studies on aerial locomotion at the lower limit of body sizes.^64^

### Limitations of the study

The 3-dimensional virtual models of the insect’s visual apparatus are reconstructed using micro-computer tomography. Compared to high-resolution scanning electron microscopy, micro-computer tomography has limited optical resolution, which may lower the accuracy of the reported optical parameters. For calculations of lens optics we only considered light refraction at the transition from air to the corneal side of the lens. Although whiteflies are not the smallest flying insect species, our data suggest a lower size limit for the optical components of the insect compound eye. To prove this finding on a broader level, future work needs to confirm our data in the smallest insect species with body sizes less than approximately 1.0 mm.

## Resource availability

### Lead contact

Further information and requests for resources and methods should be directed to the lead contact Fritz-Olaf Lehmann (fritz.lehmann@uni-rostock.de).

### Materials availability

Animals are available from the lead contact upon request.

### Data and code availability

The authors declare that the data supporting the findings of this study are available within the article.

• Data: All data reported in this paper will be shared by the lead contact upon request.
• Code: This paper uses self-developed software (Calculation.m) written in MATLAB. The download link for the software is provided in the key resources table.
• Other items: Any additional information required to understand the results are available from the lead contact upon request.

## Acknowledgments

We thank Christian Wirkner and Stephan Scholz (Institute of Biosciences, Department of General and Special Zoology, University of Rostock, Germany) for their help on micro-tomography imaging. This work was supported by the 10.13039/501100001659Deutsche Forschungsgemeinschaft (10.13039/501100001659DFG) by grant LE 905/18-1 to F.O.L.

## Author contributions

Conceptualization: F.-O.L. and T.U.; methodology: T.U. and F.-O.L.; formal analysis: T.U.; investigation: T.U.; resources: F.-O.L.; data curation: F.-O.L.; writing – original draft: T.U.; writing – review and editing: F.-O.L.; visualization: T.U.; project administration: F.-O.L. and T.U.; funding acquisition: F.-O.L.

## Declaration of interests

The authors declare no competing interests.

## STAR★Methods

### Key resources table

**Table:** 

REAGENT or RESOURCE	SOURCE	IDENTIFIER
**Chemicals, peptides, and recombinant proteins**		

Duboscq-Brasil fixative	This study	N/A
Clear adhesive	Loctite	Catalog #1KZ1103A

**Experimental models: Organisms/strains**		

*Bemisia tabaci* (wildtype)	This study	N/A

**Software and algorithms**		

Calculation.m (MATLAB)	This study	https://doi.org/10.5281/zenodo.15352423
ImageJ	ImageJ	https://imagej.net/ij/
MATLAB® 2018b	The Mathworks	https://mathworks.com
Powerpoint 2016	Microsoft	https://www.microsoft.com
CoreDRAW X6	Corel Corporation	https://www.coreldraw.com
DLTdv7	Tyson Hedrick	https://biomech.web.unc.edu/dltdv/
Vaa3D	Hanchuan Peng	https://github.com/vaa3d

**Other**		

Xradia 410 Versa μCT	Zeiss	https://www.zeiss.com/microscopy
EM CPD300 Point dryer	Leica	https://www.leica-microsystems.com
Phantom V12.1	Vision Research	https://www.phantomhighspeed.com
AF Micro Nikkor 60mm	Nikon	https://www.nikon.com
Conversion lens DCR-250	Raynox	http://www.raynox.co.jp

### Experimental model and study participant details

All experimental animals used in this study are listed in the key resources table and include both male and female organisms at unknown age. The animals stem from our laboratory stock and were initially provided by Gal Ribak, Tel Aviv University, Israel. Detailed experimental procedures are explained in the Methods Details section.

### Method details

#### Rearing of experimental animals

*Bemisia tabaci* were reared at ∼50% humidity and ∼27°C ambient temperature on sweet potato, eggplant and pumpkin plants. The growing tent was illuminated by a 75W full spectrum LED grow light (CXHome, Amazon) on a 12h day/night cycle.

#### Micro tomography

Whiteflies with unknown age were collected from their host plants by an aspirator and killed at −4°C in a freezer. We removed the abdomen and transferred the remaining body, i.e., head and thorax, to glass vials containing Duboscq-Brasil fixative.^65^ After tissue fixation at room temperature for 24 h, the animals were dehydrated for 10 min each in rising concentrations of ethanol of 70%, 80%, 90%, 96% and 98.8% (twice in each solution) and subsequently dried in a critical point dryer (LEICA EM CPD300). The bodies were then mounted and placed inside a micro-CT (ZEISS Xradia 410 Versa X-ray) at 6 mm distance from the X-ray source and 1 mm away from the 40× magnifying lens. We performed micro-CT scans at 50 kV, 8 W and 10 s exposure time per image without pixel binning. Image resolution is 0.29 μm × 0.29 μm per video pixel and images were exported as lossless TIF-stacks. During post-processing, we enhanced image contrast using ImageJ (Wayne Rasband, NIH; Figures 1 and 2).

#### Free and tethered flight essays

We measured body posture in 21 freely flying male and female whiteflies. The animals took-off from a post inside a custom-built miniature 3 cm × 1.5 cm × 2 cm flight chamber. The front of the chamber was made from a transparent glass slide while the back was a mirror tilted ∼65° toward a high-speed video camera (Phantom v12.1, Vision Research Inc.). Frame recording rate was 7000 fps and the two views on the freely flying animal were spatially calibrated using DLTdv7 software^66^ in MATLAB 2018b (The Mathworks). Due to the limited focal depth of the calibrated cameras in both views, only straight flights were selected and analyzed. On each video image, we tracked one point on the head and one between the abdomen and thorax. From these 3-dimensional data points, we calculated instantaneous body position, flight velocity and body pitch angle (Figure 3A). The abdomen tip was tracked to determine the animal’s body length. Flight duration varied from 155 to 800 video frames and data were normalized using the spline function in MATLAB to compare temporal changes in velocity and body angles (Figure 3C).

To study the potential changes in visual gaze due to head motion, we anesthetized 12 whiteflies on a ∼1°C Peltier stage and glued their prothorax to a ∼7.3 mm long tungsten rod using UV-light activated glue (Clear Glass Adhesive, Henkel Loctite). The rod was chemically etched to ∼0.1 mm tip diameter. The animals were orientated at ∼80° body pitch angle with respect to the horizontal that approximately equals mean body pitch during free flight. To separately determine head roll and pitch angles, the animals were either filmed from the front (roll, Figure 3E) or the side (pitch, Figure 3F) by a high-speed video camera equipped with a 60 mm macro lens (Nikkon), spacer rings and a macro conversion lens (DCR-250, Raynox). The camera’s frame recording rate was 7000 fps and flight initiated by gentle blowing on the animal. Instantaneous head roll angle was calculated from the position of the left and right ventral eye and pitch angle from the thorax and the dorsal section of the right eye (Figures 4E and 4F).

#### Reconstruction of optical properties

To determine optical properties of the compound eye lenses from micro-CT images, we generated three-dimensional virtual models of the whitefly head using Vaa3D software (Figure 1A).^67^ Each corneal lens is characterized by 6 three-dimensional, manually digitized, virtual points (blue, Figure 1C) indicating the lens basis and a single point indicating the lens top (red, Figure 1C). The xyz-position of these points are with respect to the head plane defined by the positions of ventral and dorsal eye sections and the ocelli. Lens diameter, lens curvature and the orientation of the major visual axis were calculated using self-written software routines in MATLAB. The software included the following steps: (1) virtual points of each lens were normalized to head width, i.e., the distance between both ocelli and then (2) rotated into a 2-dimensional plane in which the area of lens basis was maximum; (3) lens diameter was estimated from the area of a circular function fitted to the 6 virtual points (blue, Figure 1C), and (4) the orientation of visual axis of the ommatidium derived from the line through the digitized point on the lens top and the midpoint of the fitted circle. (5) In a last step, mean interommatidial angle was calculated from the angular deviation of the six visual axes that surrounded each ommatidium (ϕ_1_ - ϕ_6_, Figure 1E). To calculate the 2D projection map of whitefly ommatidia, the coordinates of six heads (*N* = 3 males, *N* = 3 females) were normalized to head width and subsequently averaged. The interommatidial angles were then calculated form the averaged eye coordinates.

#### Calculation of mean gaze depending on body angle

The three-dimensional eye coordinates from micro-CT images were further used to calculate the optical axis (visual gaze) of each ommatidium. Mean visual gaze is the sum of all gaze vectors and was separately derived for each eye section. The orientation of mean gaze in Figure 4 depends on body rotation angles about the animal’s center of mass. The latter was estimated from a silhouette cutout of the whitefly’s body and assuming no variance in tissue density within the animal body. For every rotational angle, we projected mean gaze of each eye sections onto a virtual sphere surrounding the body and expressed it in spherical coordinates as the model body rotated. This was systematically done for changes in yaw, pitch and roll and in steps of 1° angular change, respectively (Figure 4).

### Quantification and statistical analysis

The study uses self-developed software code written in MATLAB 2018b (The Mathworks) and ImageJ (National Institutes of Health, USA) to process video images, to calculate optical properties, to analyze all tethered and free flight experiments, and to perform statistical test. Data shown in Figures 1, 2, and 4 are from the right compound eye of 3 female and 3 male whiteflies. Data in Figure 3D are from 21 animals and in Figures 3E and 3F from 12 animals. Data are shown as means in Figures 1D and 2A, and as means ± standard deviation in Figure 3D and the main text. Findings in Figures 2B–2D are shown as boxplots with means (cross), medians (horizontal line), quartiles (hinges) and whiskers extending to the most extreme data point no further than 1.5 times the interquartile range away from the box. Outliers are plotted as circles. For statistical comparisons we used two-sample t tests and for graphical representations MATLAB, Microsoft Powerpoint and CorelDRAW X6 software.

## References

[1] Srinivasan M.V., Poteser M., Kral K. (1999). Motion detection in insect orientation and navigation. Vision Res..

[2] Campbell H.R. (2001). Orientation discrimination independent of retinal matching blowflies. J. Exp. Biol..

[3] Srygley R.B., Oliveira E.G. (2001).

[4] Dacke M., Nilsson D.E., Scholtz C.H., Byrne M., Warrant E.J. (2003). Insect orientation to polarized moonlight. Nature.

[5] Hesselberg T., Lehmann F. (2007). Visuo-motor learning during orientation flight of the fruit fly *Drosophila*. Comp. Biochem. Physiol. A.

[6] Götz K.G. (1987). Course-control, metabolism and wing interference during ultralong tethered flight in *Drosophila melanogaster*. J. Exp. Biol..

[7] Borst A. (2014). Fly visual course control: behaviour, algorithms and circuits. Nat. Rev. Neurosci..

[8] Götz K.G., Hengstenberg B., Biesinger R. (1979). Optomotor control of wing beat and body posture in *Drosophila*. Biol. Cybern..

[9] Hengstenberg R. (1991). Body posture, head posture and gaze movements in the fruitfly. Verh. Dtsch. Zool. Ges..

[10] Berthé R., Lehmann F.-O. (2015). Body appendages fine-tune posture and moments in freely manoeuvring fruit flies. J. Exp. Biol..

[11] Borst A., Egelhaaf M. (1989). Principles of visual motion detection. TINS (Trends Neurosci.).

[12] Franceschini N., Riehle A., Nestour A.l. (1989). Facets of vision, Stavenga, and Hardie.

[13] Zanker J.M. (1990). On the directional sensitivity of motion detectors. Biol. Cybern..

[14] Haag J., Denk W., Borst A. (2004). Fly motion vision is based on Reichardt detectors regardless of the signal-to-noise ratio. Proc. Natl. Acad. Sci. USA.

[15] Wardill T.J., Fabian S.T., Pettigrew A.C., Stavenga D.G., Nordström K., Gonzalez-Bellido P.T. (2017). A novel interception strategy in a miniature robber fly with extreme visual acuity. Curr. Biol..

[16] Taylor G.J., Hall S.A., Gren J.A., Baird E. (2020). Exploring the visual world of fossilized and modern fungus gnat eyes (Diptera: Keroplatidae) with X-ray microtomography. J. R. Soc. Interface.

[17] Currea J.P., Sondhi Y., Kawahara A.Y., Theobald J. (2023). Measuring compound eye optics with microscope and microCT images. Commun. Biol..

[18] Land M.F., Eckert H. (1985). Maps of the acute zones of fly eyes. J. Comp. Physiol..

[19] Land M.F. (1989). Facets of vision.

[20] van Hateren J.H., Hardie R.C., Rudolph A., Laughlin S.B., Stavenga D.G. (1989). The bright zone, a specialized dorsal eye region in the male blowfly Chrysomyia megacephala. J. Comp. Physiol..

[21] Lancer B.H., Evans B.J.E., Wiederman S.D. (2020). The visual neuroecology of anisoptera. Curr. Opin. Insect Sci..

[22] Meece M., Rathore S., Buschbeck E.K. (2021). Stark trade-offs and elegant solutions in arthropod visual systems. J. Exp. Biol..

[23] Stöckl A., Grittner R., Taylor G., Rau C., Bodey A.J., Kelber A., Baird E. (2022). Allometric scaling of a superposition eye optimizes sensitivity and acuity in large and small hawkmoths. Proc. Roy. Soc. B.

[24] Land M.F. (1997). Visual acuity in insects. Annu. Rev. Entomol..

[25] Makarova A.A., Meyer-Rochow V.B., Polilov A.A. (2019). Morphology and scaling of compound eyes in the smallest beetles (Coleoptera: Ptiliidae). Arthropod Struct. Dev..

[26] Fischer S., Lu Z., Meinertzhagen I.A. (2019). Three-dimensional ultrastructural organization of the ommatidium of the minute parasitoid wasp *Trichogramma evanescens*. Arthropod Struct. Dev..

[27] Polilov A.A. (2015). Small is beautiful: features of the smallest insects and limits to miniaturization. Annu. Rev. Entomol..

[28] Minelli A., Fusco G. (2019). No limits: breaking constraints in insect miniaturization. Arthropod Struct. Dev..

[29] Barlow H.B. (1952). The size of ommatidia in apposition eyes. J. Exp. Biol..

[30] Snyder A.W., Stavenga D.G., Laughlin S.B. (1977). Spatial information capacity of compound eyes. J. Comp. Physiol..

[31] Hanken J., Wake D.B. (1993). Miniaturization of body size: organismal consequences and evolutionary significance. Annu. Rev. Ecol. Syst..

[32] Polilov A.A. (2016).

[33] Sane S.P. (2016). Neurobiology and biomechanics of flight in miniature insects. Curr. Opin. Neurobiol..

[34] Reichardt W. (1987). Evaluation of optical motion information by movement detectors. J. Comp. Physiol..

[35] Egelhaaf M., Borst A. (1993). A look into the cockpit of the fly: visual orientation, algorithms, and identified neurons. J. Neurosci..

[36] Borst A., Haag J., Reiff D.F. (2010). Fly motion vision. Annu. Rev. Neurosci..

[37] Dudley R. (2002). Mechanisms and implications of animal flight maneuverability. Integr. Comp. Biol..

[38] Franz M.O., Krapp H.G. (2000). Wide-field, motion-sensitive neurons and matched filters for optic flow fields. Biol. Cybern..

[39] Sane S.P., Dieudonné A., Willis M.A., Daniel T.L. (2007). Antennal mechanosensors mediate flight control in moths. Science.

[40] Deora T., Singh A.K., Sane S.P. (2015). Biomechanical basis of wing and haltere coordination in flies. Proc. Natl. Acad. Sci. USA.

[41] Wootton R.J., Newman D.J.S. (1979). Whitefly have the highest contradiction frequencies yet recorded in non-fibrillar flight muscles. Nature.

[42] Isaacs R., Willis M.A., Byrne D.N. (1999). Modulation of Whitefly take-off and flight orientation by wind speed and visual cues. Physiol. Ecol..

[43] Isaacs R., Byrne D.N. (1998). Aerial distribution, flight behaviour and eggload: their inter-relationship during dispersal by the sweetpotato whitefly. J. Anim. Ecol..

[44] Stukenberg N., Poehling H.M. (2019). Blue–green opponency and trichromatic vision in the greenhouse whitefly (Trialeurodes vaporariorum) explored using light emitting diodes. Ann. Appl. Biol..

[45] Coombe P.E. (1982). Visual behaviour of the greenhouse whitefly, Trialeurodes vaporariorum. Physiol. Entomol..

[46] Vaishampayan S.M., Kogan M., Waldbauer G.P., Woolley J.T. (1975). Spectral specific responses in the visual behavior of the greenhouse whitefly, Trialeurodes vaporariorum (Homoptera: Aleyrodidae). Entomol. Exp. Appl..

[47] Calvert L.A., Cuervo M., Arroyave J.A., Constantino L.M., Bellotti A., Frohlich D. (2001). Morphological and mitochondrial DNA marker analyses of whiteflies (Homoptera: Aleyrodidae) colonizing cassava and beans in Colombia. Ann. Entomol. Soc. Am..

[48] Mellor H.E., Bellingham J., Anderson M. (1997). Spectral efficiency of the glasshouse whitefly *Trialeurodes vaporariorum* and *Encarsia formosa* its hymenopteran parasitoid. Entomol. Exp. Appl..

[49] Blagodatski A., Kryuchkov M., Sergeev A., Klimov A.A., Shcherbakov M.R., Enin G.A., Katanaev V.L. (2014). Under-and over-water halves of Gyrinidae beetle eyes harbor different corneal nanocoatings providing adaptation to the water and air environments. Sci. Rep..

[50] Horridge G.A., McLean M. (1978). The dorsal eye of the mayfly *Atalophlebia* (Ephemeroptera). Proc. Roy. Soc. Lond. B.

[51] Gonzalez-Bellido P.T., Wardill T.J., Juusola M. (2011). Compound eyes and retinal information processing in miniature dipteran species match their specific ecological demands. Proc. Natl. Acad. Sci. USA.

[52] Makarova A., Polilov A., Fischer S. (2015). Comparative morphological analysis of compound eye miniaturization in minute Hymenoptera. Arthropod Struct. Dev..

[53] Buschbeck E.K. (2005). The compound lens eye of Strepsiptera: morphological development of larvae and pupae. Arthropod Struct. Dev..

[54] Menzel J.G., Wunderer H., Stavenga D.G. (1991). Functional morphology of the divided compound eye of the honeybee drone (*Apis mellifera*). Tissue Cell.

[55] Jia L.-P., Liang A.-P. (2015). Fine structure of the compound eyes of *Callitettix versicolor* (Insecta: Hemiptera). Ann. Entomol. Soc. Am..

[56] Meyer-Rochow V.B. (1972). The eyes of *Creophilus erythrocephalus* F. and *Sartallus signatus sharp* (Staphylinidae: Coleoptera) Light-interference-scanning electron-and transmission electron microscope examinations. Z. Zellforsch. Mikrosk. Anat..

[57] Heisenberg M., Wolf R. (1984).

[58] Kirschfeld K. (1974). The absolute sensitivity of lens and compound eyes. Z. Naturforsch. C Biosci..

[59] Carlson S.D., Chi C. (1974). Surface fine structure of the eye of the housefly (*Musca domestica*): ommatidia and lamina ganglionaris. Cell Tissue Res..

[60] Fabian S.T., Sumner M.E., Wardill T.J., Rossoni S., Gonzalez-Bellido P.T. (2018). Interception by two predatory fly species is explained by a proportional navigation feedback controller. J. R. Soc. Interface.

[61] Mronz M., Lehmann F.-O. (2008). The free flight response of *Drosophila* to motion of the visual environment. J. Exp. Biol..

[62] Beatus T., Guckenheimer J.M., Cohen I. (2015). Controlling roll perturbations in fruit flies. J. R. Soc. Interface.

[63] Fischer S., Müller C.H.G., Meyer-Rochow V.B. (2011). How small can small be: the compound eye of the parasitoid wasp *Trichogramma evanescens* (Westwood, 1833)(Hymenoptera, Hexapoda), an insect of 0.3-to 0.4-mm total body size. Vis. Neurosci..

[64] Farisenkov S.E., Kolomenskiy D., Petrov P.N., Engels T., Lapina N.A., Lehmann F.-O., Onishi R., Liu H., Polilov A.A. (2022). Novel flight style and light wings boost flight performance of tiny beetles. Nature.

[65] Barbosa P., Berry D., Kary C.K. (2014).

[66] Hedrick T.L. (2008). Software techniques for two- and three-dimensional kinematic measurements of biological and biomimetic systems. Bioinspir. Biomim..

[67] Peng H., Bria A., Zhou Z., Iannello G., Long F. (2014). Extensible visualization and analysis for multidimensional images using Vaa3D. Nat. Protoc..

